# Alumni Association, American Veterinary College

**Published:** 1891-04

**Authors:** W. L. LaBaw

**Affiliations:** Secretary, Pro Tem.


*Alumni Association, American Veterinary College.*—A meeting of the Alumni Association of the American Veterinary College, was held in the lecture room of the college on March 18, 1891. Dr. Hoskins in the chair. The meeting was called to order at 11 A.M. Dr. LaBaw was appointed Secretary Pro Tem., in absence of Dr. Sellers.

On calling the roll sixteen were present. The minutes of the previous meeting were read and approved. The minutes of the Executive Committee of '90 were read and approved. Report of Executive Committee, Dr. Birdsall reported that the supper was to be held at the Brower House, cor. 28th St., and Broadway, and that the graduating class of '91 would unite with them. Dr. Birdsall further reported that the Alumni prizes consisted of five (5) standard medical books, to be given to the student passing the second highest examination.

Dr. LaBaw presented a bill from Sabiston & Murry, for books and supper tickets. Bill approved and secretary authorized to pay the same from assessment funds.

Dr. Coates reported on the present prospect of a new building. He said although there was still nothing definite or certain about the erection of a new building, yet a certain amount of progress had been made in the consummation of plans.

One plan for paying the debt on the lots $30,000, still needed aid to the amount of $7,000, to make it a success.

He had also found a man who was willing to erect the buildingand rent it to the college, giving the college the privilege of buying it in a certain number of years.

Dr. Coates was inclined to favor this latter plan and create a sinking fund, the money thus raised to be placed with a trust company and to be used for no other purpose than the purchase of a college building. A suggestion to sell the lots now held by the college was offered; but was objected to on the ground that they would not clear the incumberance.

Resolutions were passed on the death of Dr. Lathrop.

Under new business a motion was made requesting the Executive Committee to endeavor to call the next meeting in the afternoon instead of morning. Seconded and carried.

Then came the election of officers.

The following members were elected to office for the ensuing year:

Dr. W. H. Hoskins, President, Philadelphia, Pa.; (re-elected), Dr. Lowe, Vice-President, Paterson, N. J., in place of Dr. H. Birdsall; Dr. Ackerman, Secretary, Brooklyn, N. Y., in place of Dr. A. T. Sellers; Dr. Strange, Treasurer, New York City, N. Y., (re-elected); Dr. Birdsall, Librarian, New York City, N. Y., in place of Dr. G. S. Lathrop, deceased.

Regrets expressed upon the death of Dr. W. J. Mitchell.

W. L. LaBaw, *Secretary*, Pro Tem.

